# Comparison of High- vs. Low-Responders Following a 6-Month XC Ski-Specific Training Period: A Multidisciplinary Approach

**DOI:** 10.3389/fspor.2020.00114

**Published:** 2020-09-08

**Authors:** Rune Kjøsen Talsnes, Roland van den Tillaar, Xudan Cai, Øyvind Sandbakk

**Affiliations:** ^1^Meråker High School, Trøndelag County Council, Steinkjer, Norway; ^2^Department of Sports Science and Physical Education, Nord University, Bodø, Norway; ^3^School of Physical Education and Sport Training, Shanghai University of Sport, Shanghai, China; ^4^Olympic Games Preparation Office, Chinese Olympic Committee, Beijing, China; ^5^Centre for Elite Sports Research, Department of Neuromedicine and Movement Science, Norwegian University of Science and Technology, Trondheim, Norway

**Keywords:** cross-country skiing, maximal oxygen uptake, endurance training, training load, coaching

## Abstract

Individual training responses among endurance athletes are determined by a complex interplay between training load, recovery and genetic influence. The present study used a multidisciplinary approach to compare high- and low-responders following a 6-month training period in endurance athletes transferring to cross-country (XC) skiing. Twenty-three endurance-trained athletes (14 runners and 9 rowers/kayakers; 14 men and 9 women) were classified as high (*n* = 9) or low-responders (*n* = 11) based on pre- to post changes in treadmill running, roller-ski skating and double-poling ergometry performances following 6-months of standardized XC ski-specific training. Physiological and technical capacities during these same modes were monitored pre and post. In addition, training volume, intensity, mode and session rating of perceived exertion (sRPE) training load were quantified daily. Finally, qualitative interviews of the athlete's personal coaches were performed after the intervention. There were no differences between groups with respect to physiological baseline characteristics. High-responders improved maximum oxygen uptake (VO_2max_) in treadmill running (5.5 ± 7.0% change from pre- to post) as well as peak oxygen uptake (VO_2peak_; 7.3 ± 7.0%) and power output at 4 mmol·L^−1^ (37.7 ± 28.2%) treadmill roller-ski skating which differed from a corresponding non-significant change in low-responders (−1.2 ± 3.6%, −2.7 ± 3.7% and 8.2 ± 12.5%; all *P* ≤ 0.05). VO_2peak_ in double-poling ergometry did not change in any group, whereas gross efficiency and cycle length in roller-ski skating improved in both groups. High-responders performed greater training loads (weekly load: 3825 ± 1013 vs. 3228 ±.748 and load/volume ratio: 4.9 ± 0.6 vs. 4.2 ± 0.5; both *P* ≤ 0.05) and had lower incident of injury/illness (5 ± 3 vs. 10 ± 5 days; *P* = 0.07). Their coaches highlighted high motivation to train and compete, together with the ability to build a strong coach-athlete relationship, to separate high- from low-responders. In conclusion, high-responders to 6-months of standardized XC ski-specific training demonstrates greater improvement in maximal/peak aerobic capacity, which was coincided by higher training loads, greater perceived effort during sessions and lower incidents of injury and illness in comparison to their lower-responding counterparts. Possibly, the higher motivation and stronger coach-athlete relationships in high-responders contributed to more individually optimized training and recovery routines, and thereby more positive performance-development.

## Introduction

Individual training responses among endurance athletes are determined by a complex interplay between training load (i.e., volume, intensity and frequency) and the subsequent recovery (e.g., sleep, nutrition and non-training daily stressors), in which pre-training status and genetic influence plays additional roles (Mann et al., 2014). Accordingly, training responses to standardized training programs demonstrates large inter-individual variations with individuals represented in both ends of the response range, a phenomenon often referred to as “high- and low-responders” (Mann et al., 2014). The main principles of elite endurance training are relatively similar across athletes and endurance sports (Stöggl and Sperlich, 2015); however, individual manipulation of training load is required to optimize an individual athlete's training response and avoid negative outcomes such as injury, illness, non-functional overreaching and/or overtraining (Halson, 2014; Mujika, 2017). Although enhanced understanding of underlying factors for individual differences in the tolerance and response to training is of uttermost importance (Mann et al., 2014; Mujika, 2017), no previous study has examined factors differentiating high- from low-responders following long-term periods of standardized endurance training.

Cross-country (XC) skiers perform high loads of endurance training and achieve some of the highest reported maximum oxygen uptakes (VO_2max_) in the scientific literature (Holmberg, 2015; Sandbakk and Holmberg, 2017). Furthermore, high peak oxygen uptakes (VO_2peak_) within sport-specific exercise modes, together with well-developed efficiency and the ability to produce long cycle lengths are considered important determinants of performance in XC skiing (Sandbakk and Holmberg, 2017). Accordingly, the training of elite XC skiers targets a concurrent development of the abovementioned capacities and include around 750–950 annual training hours, of which ~85–90% is endurance and ~10–15% is speed and strength training. The endurance training follows a typical polarized intensity distribution consisting of large amounts of low-intensity training (LIT) and moderate amounts of high-intensity training (HIT) (Sandbakk and Holmberg, 2017). Although the physiological capacities and training characteristics of successful XC skiers (Sandbakk and Holmberg, 2017; Solli et al., 2017), and differences among their less successful counterparts (Sandbakk et al., 2011, 2016), are well-established in the scientific literature, the understanding of accompanying factors influencing individual performance-adaptations on the journey toward excellence is limited.

Aiming for XC skiing success at the Beijing Winter Olympic Games in 2022, China has developed a talent transfer program (also referred to as athlete transfer program), where athletes from various summer sports are transferred to XC skiing by utilizing state-of-the-art training and coaching methods (Sandbakk and Holmberg, 2017). Although talent transfer programs are commonly used in sport practice, characteristics of the most sucessfull athletes following such initiatives are currently understudied in the scientific litterature. Here, we expect particularly large individual responses to training due to the athletes various pre-training history and sport background. Therefore, the current study used a multidisciplinary approach to compare high- and low-responders following a 6-month training period in endurance athletes transferring to XC skiing.

## Methods

### Participants

The participants consisted of 24 Chinese endurance transfer athletes, in which 1 was excluded due to missing training and training load data (14 runners, 7 kayakers and 2 rowers; 14 men and 9 women; age 19 ± 2 y; body-mass 66.7 ± 10 kg; body-height 175.9 ± 10.6 cm; body-mass index 21.5 ± 1.6). All athletes were selected from the group of the second-best athletes in their respective sports in China and had trained professionally for this sport over several years. Therefore, they were given the opportunity to transfer from their summer sport to XC skiing while aiming for participation in the Beijing 2022 Olympic Games (this information is based on verbal communication with members of the Chinese Olympic Committee).

### Ethics Statement

The Regional Committee for Medical and Health Research Ethics waives the requirement for ethical approval for such studies. Therefore, the ethics of the study is done according to the institutional requirements and approval for data security and handling was obtained from the Norwegian Center for Research Data. Prior to the data collection, all participants provided written informed consent to voluntarily take part in the study. The participants were informed that they could withdraw from the study at any point in time without providing a reason for doing so.

### Study Design

After an initial 3.9 ± 1.9-month introduction to XC roller-skiing in China, the athletes completed a 6-month training period (talent transfer program) of XC ski-specific training in Norway from November 2018 to May 2019. Athletes were classified as high (*n* = 9) or low-responders (*n* = 11) based on pre- to post changes in laboratory performance in treadmill running, roller-ski skating and double-poling ergometry performances following the training period. Physiological and technical capacities during these same modes, as well as upper-body, one-repetition maximum-strength (1RM) were monitored pre and post. In addition, training volume, intensity and mode were logged daily, and session rating of perceived exertion (sRPE) training load quantified. Finally, qualitative interviews of the athletes' seven personal coaches were conducted directly after the training period to provide an understanding of possible psychological and/or sociological factors associated with the training and recovery process.

### Performance Index

In order to classify high- from low-responders to the 6-month training period, a performance index was developed based on pre- to post changes in peak treadmill speed (V_peak_) during a VO_2max_ test while treadmill running and a VO_2peak_ test while treadmill roller-ski skating, in addition to average power output in a 5-min and 30-s performance test double-poling ergometry. The relative (%) changes in these four performance measures were further summated by the following formula:

Performance index = %ΔV_peak_ treadmill running (m·s^−1^) + %ΔV_peak_ treadmill roller-ski skating (m·s^−1^) + %ΔPower output 5-min test double-poling (w) + %ΔPower output 30-s test double-poling (w).

**Figure 1:**
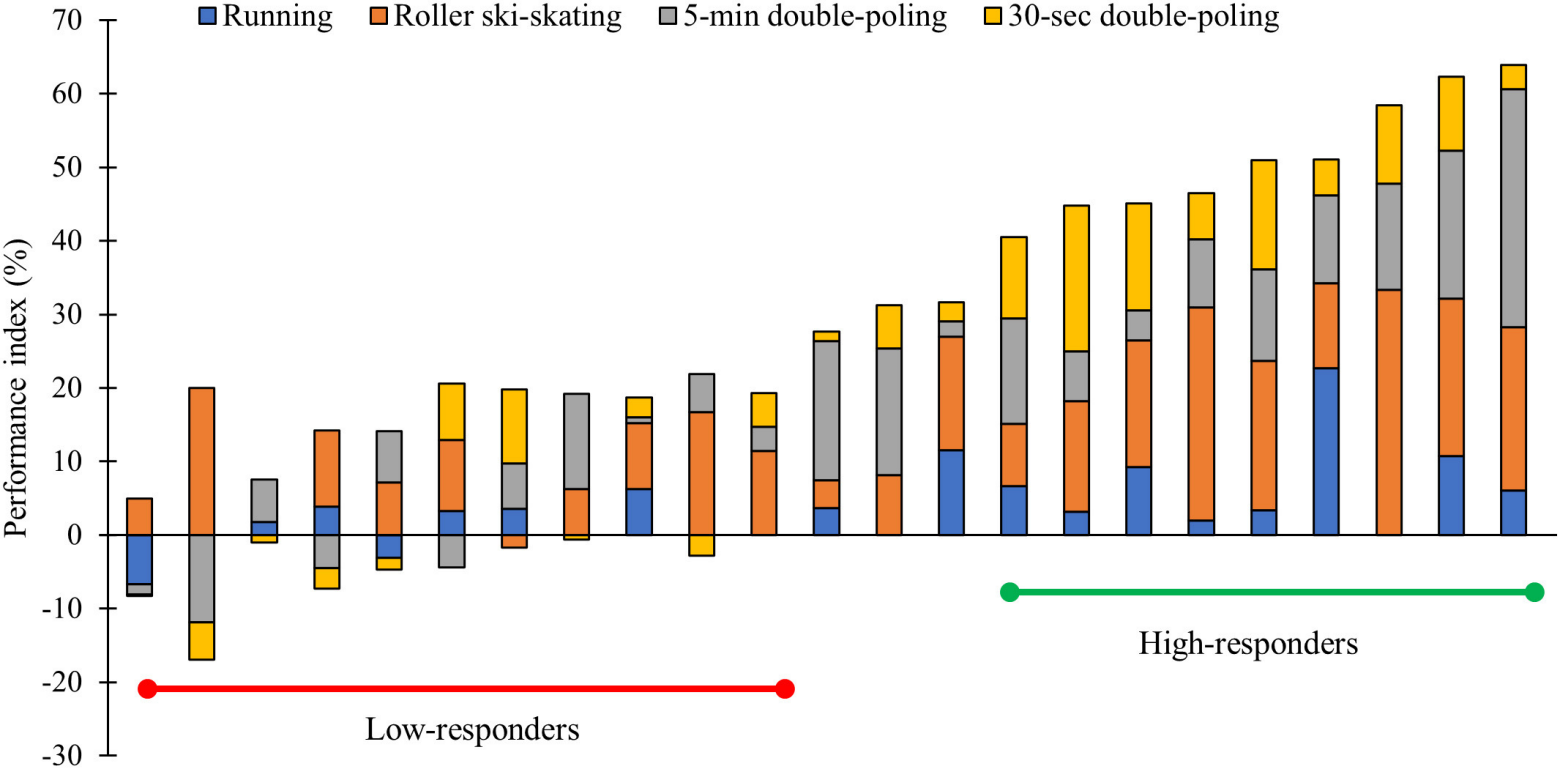
Frequency distribution of the individual response magnitude (performance index) based on pre- to post changes in peak speed during a VO_2max_ test treadmill running and VO_2peak_ test treadmill roller-ski skating in addition to average power output during a 5-min and 30-s performance test double-poling ergometry in 23 endurance transfer athletes following a 6-month XC ski-specific training period.

This resulted in a performance index ranging from −3 to 62% between athletes, with cutoffs set at >40% and <20% for classifying high- and low-responders, respectively. Hence, 9 athletes were defined as high-responders and 11 athletes as low-responders, whereas 3 athletes with a performance index in-between the two cutoffs were excluded from further analyses to establish distinct group differences. The individual response magnitude in performance index among all athletes are displayed in Figure 1. High-responders consisted of 8 men and 1 woman, whereas low-responders consisted of 6 men and 5 women, respectively. Furthermore, 8 athletes in the high-responder group had a previous sport background from running (i.e., long or middle-distance) and 1 from kayaking. In the low-responder group, 5 athletes had a previous sport background from running, whereas 2 and 4 had a sport background from rowing and kayaking, respectively.

### Laboratory Test Protocols and Measurements

The athlete's performance, physiological and technical capacities in treadmill running, treadmill roller-ski skating and double-poling ergometry as well as upper-body 1RM strength were measured during a test week conducted over a 5-day period including 3 days of testing and 2 days of easy training in between. The easy training days consisted of one session of 90-min low-intensity roller-skiing or skiing. Test day 1 consisted of physiological and performance testing during treadmill running followed by upper-body 1RM strength tests. Test day 2 consisted of physiological, technical and performance testing during treadmill roller-ski skating, whereas on test day 3, athletes performed physiological and performance testing using double-poling ergometry. Prior to all tests, the athletes completed a standardized 10-min low-intensity warm-up by running on a treadmill, being instructed to keep an exercise intensity corresponding to 3 on a 1–10-point rating of perceived exertion (RPE) scale.

### Treadmill Running Tests (Test Day 1)

Physiological and performance testing in treadmill running was conducted using protocols developed by the Norwegian Top Sport Center as previously described (Ingjer, 1992; Tønnessen et al., 2015). Submaximal lactate profile testing was considered complete when the athletes reached a blood lactate value of ≥4 mmol·L^−1^. After a 5-min recovery, the athletes conducted an incremental test to determine VO_2max_ and performance measured as V_peak_ calculated according to Sandbakk et al. (2011).

### Upper-Body Strength Tests (Test Day 1)

After−20-min of recovery, all athletes were tested for 1RM upper-body strength in the ski-specific exercises, seated pull-down and triceps press, using protocols described in detail by Losnegard et al. (2011). All 1RM testing was conducted using the same equipment with identical equipment positioning for each athlete, and all tests were supervised by the same test leader who gave verbal feedback to ensure proper technique. All athletes were familiar with the exercises before testing.

### Treadmill Roller-Skiing Tests (Test Day 2)

Initially, submaximal lactate profile testing was performed at a constant speed (2.5 m·s^−1^) and starting incline of 1° using a graded protocol, including 3–6 periods of 5-min stages with a stepwise increase in workload (1°) and 60-s recovery in between each stage. Heart rate was defined as the average of the last 30-s of each stage and RPE and blood lactate values were determined directly after completing each stage. At a given submaximal workload (3°), VO_2_ and video recordings were included between the third and fifth minute of the stage to determine cycle characteristics as well as calculations of submaximal oxygen cost (O_2_-cost) and efficiency. Gross efficiency was used as a measure of efficiency and defined as the ratio of work rate and metabolic rate as described by Sandbakk et al. (2010). The submaximal test was considered complete when the athletes reached a blood lactate value of ≥4 mmol·L^−1^. After a 5-min recovery period, VO_2peak_ and performance-measured V_peak_ testing were performed as an incremental test with a starting incline and speed of 4° and 2.5 m·s^−1^, respectively. The incline was kept constant, while the speed was subsequently increased by 0.28 m·s^−1^ every 60 s until voluntary exhaustion. Respiratory variables and heart rate were measured continuously, and VO_2peak_ was defined as the average of the two highest and consecutive 30-s measurements. Peak heart rate was defined as the highest 5-s heart rate measurement during the test, whereas RPE was determined directly after, and blood lactate values approximately 1 min after, completing the test. Power at 4 mmol·L^−1^ was calculated using linear interpolation.

### Double-Poling Ergometer Tests (Test Day 3)

Initially, a 3-min specific warm-up (RPE = 4) double-poling ergometry was performed following the 10-min standardized warm-up protocol. Thereafter, all athletes conducted a modified 30-s Wingate test and a 5-min self-paced performance test with a 5-min recovery period in between using protocols similar to those in previous studies of XC skiing (Hegge et al., 2015, 2016). The athletes were instructed to perform the 30-s Wingate test as all-out, whereas based on previous training using double-poling ergometry, instructions for an even, maximal pacing were given prior to the 5-min test to prevent “overpacing.”

### Monitoring, Registration, and Systematization of Training

During the 6-month training period, all athletes followed standardized XC ski-specific training although each athlete had a personal coach who helped them to daily adjust their training to ensure optimized training load and adaptations for each individual athlete. A typical training week normally consisted of two daily training sessions (i.e., morning session at 09:00 AM and afternoon session at 04:00 PM). In addition, a third session was conducted early in the morning (i.e., 08:00 AM), named “XC skiing drills” and had a duration of approximately 30-min with focus on developing fundamental XC ski-specific skills (e.g., coordination, balance and stability). Day-to-day training data was registered and systematized in detail for all athletes according to the modified session-goal approach (Sylta et al., 2014). Days and/or sessions where the athletes were not able to follow standardized training due to illness or injury were verified by a medical doctor and registered. Training recorded for each session included total training time distributed across training forms (i.e., endurance, strength, and speed), exercise-mode (i.e., skiing, roller-skiing, running), and intensity zones as describes elsewhere (Tønnessen et al., 2014; Solli et al., 2017). Distribution of endurance-training intensity were reported using a three-zone scale (LIT, MIT; moderate intensity training and HIT) based on the ventilatory changes corresponding to the first-and second-lactate threshold (Seiler and Kjerland, 2006). Endurance training sessions were further divided into di□erent parts (e.g., warm-up, intervals, and cool-down). For MIT and HIT sessions performed as intervals, time in the given intensity zone was registered from the beginning of the first interval to the end of the last interval. In addition, strength and speed training were registered from the start to the finish of that separate part of the session (e.g., strength, speed, plyometrics). All training data was registered and systematized by coaches and researchers contributing to the project.

After completing each training session, athletes reported their sRPE on a 1–10 scale to a researcher who was systematizing these data. Training load was calculated by multiplying the athletes sRPE by the duration of the session in minutes, and is regarded a valid estimate for quantifying the internal load (Foster, 1998). Training load for all sessions was further summated to determine each athlete weekly training load for the entire 6-month training period. In addition, weekly training load was divided by weekly training volume (total training time), providing a load/volume ratio or weekly average RPE used to assess the relationship between perceived effort during sessions and training volume.

### Interviews

To gain a more comprehensive understanding of the multidisciplinary factors contributing to individual training responses, semi-structured interviews were conducted with the seven Norwegian coaches who worked as the athletes' personal coaches during the 6-month training period. Each coach was fully employed to follow up 3–5 athletes and could therefore closely track their development process (e.g., through daily training sessions, technical instructions, meetings and by writing monthly reports of the athlete's development). In this study, we examined their views on training load and quality, rate of injury and illness, physiological and technical development, as well as psychological and/or sociological differences between high and low performance-responders among their group of athletes. All interviews were conducted face-to-face and tape-recorded in their entirety before being transcribed and analyzed. For these data, a content analysis was conducted independently by two researchers. All coaches volunteered for the study and signed an informed consent prior to their participation.

### Statistical Analyses

All data are reported as means ± standard deviations (SD). Assumption of normality was tested with a Shapiro-Wilk test in combination with visual inspection of data. Between group differences in baseline and training characteristics were tested using an independent-samples *t*-test in combination with Mann-Whitney U test. To test for significant pre- to post changes within groups, paired-samples *t*-test were applied and Wilcoxon signed rank test when data deviated from normally distribution. Pre- to post changes between-groups were assessed using a univariate General Linear Model (GLM) (analysis of covariance (ANCOVA) adjusted for baseline (pre) values. Effect size (ES) of from pre- to post changes within and between groups were calculated according to Cohens d, and interpretations of the magnitude were as follows: 0–0.2 = trivial, 0.2–0.6 = small, 0.6–1.2 = moderate, 1.2–2.0 = large, and >2 = very large (Hopkins et al., 2009). For all comparisons, statistical significance was set at an alpha level of P < 0.05. All data analyses were conducted using SPSS 26.0 (SPSS Inc, Chicago, IL, United States) and Excel 2016 (Microsoft Corporation, Redmond, Washington, United States).

## Results

### Baseline Characteristics and Development of Performance Indicators

The calculated performance index was higher in high- (51.5 ± 7.8%) compared to low-responders (12.0 ± 7.5%; *P* ≤ 0.001). Either of the four performance tests differed between high- and low-responders at baseline (Table 1), whereas pre- to post improvements were significantly larger for V_peak_ treadmill running, V_peak_ treadmill roller-ski skating and average power output in the 5-min and 30-s performance test double-poling ergometry in high- (7.1 ± 6.4%, 20.0 ± 7.2, 14.5 ± 7.5, and 10.3 ± 5.0%, respectively) compared to low-responders (0.8 ± 3.4%, 8.3 ± 5.8, 1.7 ± 6.6, and 1.2 ± 4.9%, respectively; Table 1; all *P* ≤ 0.05). High- and low-responders did not differ significantly in age (18.6 ± 1.4 vs. 19.7 ± 1.9 yrs.), body-mass (64.7 ± 5.3 vs. 68.4 ± 12.4 kg) or body-height (175.4 ± 4.3 vs. 176.4 ± 13.7 cm) at baseline. No significant changes in body-mass or body-height was observed from pre- to post neither within nor between groups.

**Table 1:** Performance, physiological and technical capacities (mean ± SD) in treadmill running, treadmill roller-ski skating and double-poling ergometry as well as upper-body 1RM strength in high and low-responders at pre and post of a 6-month XC ski-specific training period.

	**High-responders**		**Low-responders**		**Pre-post**
	**Pre**	**Post**	**Pre**	**Post**	**ES^**a**^**
**Treadmill running**					
V_peak_ (m·s^−1^)	4.15 ± 0.50	4.42 ± 0.33**	3.98 ± 0.33	4.00 ± 0.28^#^	1.36
VO_2max_ (L·min^−1^)	4.32 ± 0.54	4.52 ± 0.50^*^	4.34 ± 1.04	4.25 ± 0.90^#^	1.20
VO_2max_ (mL·min^−1^·kg^−1^)	67.0 ± 7.6	70.4 ± 4.8^*^	63.0 ± 6.6	62.2 ± 6.5^#^	1.23
Submaximal speed 4 mmol·L^−1^ (m·s^−1^)	2.77 ± 0.24	2.84 ± 0.23	2.57 ± 0.27	2.56 ± 0.26	0.54
**Treadmill roller-ski skating**					
V_peak_ (m·s^−1^)	3.88 ± 0.21	4.65 ± 0.28***	3.91 ± 0.28	4.23 ± 0.27**^#^	1.71
Power VO_2peak_ (W)	238 ± 24	283 ± 22**	255 ± 56	267 ± 50**^#^	1.13
VO_2peak_ (L·min^−1^)	4.00 ± 0.39	4.26 ± 0.41**	4.13 ± 0.88	4.01 ± 0.87**^#^	1.84
VO_2peak_ (mL·min^−1^·kg^−1^)	62.0 ± 5.8	66.3 ± 5.8**	60.2 ± 5.7	58.5 ± 5.6**^#^	1.80
Submaximal power 4 mmol·L^−1^ (W)	129 ± 30	173 ± 30**	154 ± 37	165 ± 41^*^^#^	1.53
Submaximal O_2_-cost (L·min^−1^)	2.87 ± 0.19	2.74 ± 0.19**	2.95 ± 0.57	2.82 ± 0.53**	0.06
Submaximal respiratory exchange ratio	0.95 ± 0.05	0.90 ± 0.03**	0.95 ± 0.04	0.94 ± 0.05^#^	0.97
Submaximal heart rate (beats·min^−1^)	166 ± 12	153 ± 9**	161 ± 10	159 ± 11^#^	1.25
Submaximal blood lactate (mmol·L^−1^)	3.7 ± 1.1	2.1 ± 0.7**	3.2 ± 0.9	2.5 ± 0.7**	0.83
Submaximal RPE (1-10)	3.1 ± 0.6	2.7 ± 0.7**	3.5 ± 0.9	3.2 ± 0.5	0.00
Submaximal gross efficiency (%)	12.5 ± 1.1	13.3 ± 0.6**	13.0 ± 1.0	13.5 ± 0.8**	0.23
Submaximal cycle length (m)	5.32 ± 0.34	6.05 ± 0.47**	4.97 ± 0.34^†^	5.65 ± 0.37**	0.17
Submaximal cycle rate (Hz)	0.47 ± 0.03	0.42 ± 0.03**	0.51 ± 0.03	0.44 ± 0.03**	0.12
**Double-poling ergometry**					
Power output 5-min test (W)	193 ± 22	219 ± 20**	208 ± 53	212 ± 40^#^	1.58
Peak power output 5-min test (W)	274 ± 40	292 ± 40	274 ± 70	266 ± 67	0.36
Power output 30-sec test (W)	333 ± 35	368 ± 47**	352 ± 110	353 ± 105^#^	2.13
Peak power output 30-sec test (W)	394 ± 43	441 ± 56**	416 ± 131	443 ± 167	0.47
VO_2peak_ (L·min^−1^)	3.90 ± 0.40	4.05 ± 0.25	3.85 ± 1.06	3.90 ± 0.98	0.45
VO_2peak_ (mL·min^−1^·kg^−1^)	60.7 ± 7.3	63.1 ± 5.6	55.6 ± 8.0	56.5 ± 6.1	0.37
**1RM upper-body strength**					
Seated pull-down exercise (kg)	57.0 ± 5.7	65.9 ± 8.3**	60.5 ± 14.4	65.5 ± 13.6**	0.84
Triceps-press exercise (kg)	61.3 ± 6.9	68.8 ± 6.0**	63.0 ± 13.1	67.0 ± 14.1**	0.78

*V_peak_, peak treadmill speed; VO_2max_, maximum oxygen uptake; VO_2peak_, peak oxygen uptake; RPE, rating of perceived exertion (1–10)*.

† *Significant difference between groups at baseline (pre)*.

* Significant pre- to post change within groups (*P < 0.05, **P < 0.01, ***P < 0.001).

# *Significant difference in pre- to post change between groups (P < 0.05)*.

a *ES of pre- to post change between groups calculated according to Cohens d*.

### Development of Physiological and Technical Capacities

**Figure 2:**
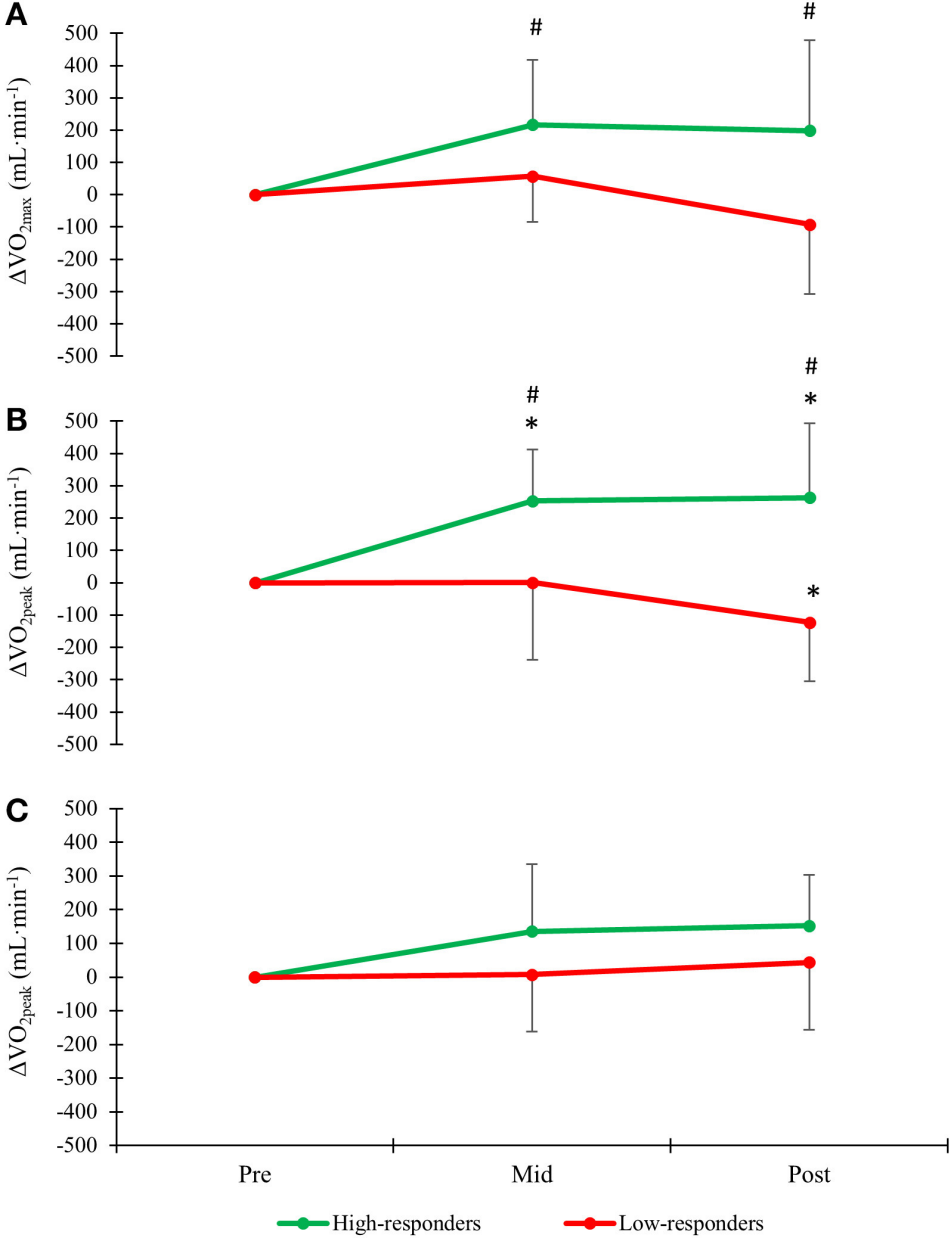
Changes in **(A)** VO_2max_ treadmill running and **(B)** VO_2peak_ treadmill roller-ski skating and **(C)** VO_2peak_ double-poling ergometry in high- and low-responders following a 6-month XC-ski specific training period. *Significant pre- to post change within groups (*P* ≤ 0.05) ^#^Significant difference in pre- to post change between groups (*P* ≤ 0.05).

Comparisons of physiological and technical capacities from pre- to post between high- and low-responders are displayed in Table 1 and Figure 2. No significant differences in VO_2max/peak_ or any other physiological measures were significantly different between groups at baseline (Table 1). Both absolute and body-mass normalized VO_2max_ treadmill running improved from pre- to post in high-responders (5.0 ± 6.8% and 5.5 ± 7.0%) which differed from a small reduction in low-responders (−1.3 ± 4.4 and −1.2 ± 3.6%; both *P* ≤ 0.05: Figure 2A). In treadmill roller-ski skating, absolute and body-mass normalized VO_2peak_ improved from pre- to post in high-responders (6.8 ± 6.1% and 7.3 ± 7.0) also differing from a corresponding reduction among low-responders (−2.8 ± 4.1% and −2.7 ± 3.7%; *P* ≤ 0.05; Figure 2B). VO_2peak_ double-poling ergometry did not change significantly pre- to post neither within nor between groups (Figure 2C).

During submaximal roller-ski skating at the same absolute speed, pre- to post changes in gross efficiency and cycle length did not differ significantly between groups, although both high- and low-responders showed within-group improvements (0.7 ± 1.0%-points vs. 0.5 ± 0.7%-points and 13.9 ± 8.1% vs. 13.9 ± 6.8%, respectively; Table 1; all *P* ≤ 0.05). Pre- to post reductions in O_2_-cost, blood lactate values and RPE did not differ between high- (−4.6 ± 5.4%, −1.6 ± 1.1 mmol·L^−^ and −0.4 ± 0.5 points, respectively) and low-responders (−4.0 ± 5.1%, −1.6 ± 0.8 mmol·L^−1^ and −0.4 ± 1.0 points, respectively; Table 1). However, submaximal heart rate, respiratory exchange ratio and power output at 4 mmol·L^−1^ improved in high-responders (-12 ± 11 beats·min^−1^, −0.05 ± 0.05 and 37.7 ± 28.2%; all *P* ≤ 0.01), which differed from a non-significant improvement among low-responders (−2 ± 6 beats·min^−1^, −0.01 ± 0.03 and 8.2 ± 12.5%; Table 1; all *P* ≤ 0.05). Changes in upper-body 1RM maximum-strength did not differ significantly between the two groups, but improved both for high- and low-responders in seated pull-down (15.7 ± 9.4% vs. 9.3 ± 6.5%; *P* ≤ 0.05) and triceps-press (12.8 ± 9.4% vs. 6.6 ± 6.3%; *P* ≤ 0.05).

### Training Characteristics

Comparisons of training characteristics between high- and low-responders during the 6-month XC ski-specific training period are displayed in Table 2. The groups did not differ in the number of rest days during the training period, but low-responders showed lower compliance to training, with on average 5 more days of reported injury and/or illness compared to high-responders (*P* = 0.07). Hence, high-responders performed 6% more total training (*P* ≤ 0.05) and completed more sessions than low-responders (*P* = 0.08). The larger total training time in high-responders consisted of 4% more endurance training distributed as 4, 8, and 8% more LIT, MIT, and HIT, respectively (all *P* ≤ 0.05; Table 2), compared to low-responders. With regards to training mode, differences in endurance training were mainly due to 6 and 7% more time running and classical skiing among high-responders (both *P* ≤ 0.05). No differences in the amount of strength and speed training performed were found between groups, but high-responders tended to use extra time of XC skiing drills (15% difference, *P* = 0.09).

**Table 2:** Training characteristics (mean ± SD) in high- and low-responders during a 6-month XC ski-specific training period.

	**High-responders**	**Low-responders**
**Total training**		
Total (h)	363 ± 11	344 ± 23^*^
Total (sessions)	311 ± 15	290 ± 30
Rest (days)	22 ± 1	22 ± 2
Injury/illness (days)	5 ± 3	10 ± 5
**Training forms**		
Endurance (h)	271 ± 6	259 ± 14^*^
Strength (h)	38 ± 1	37 ± 2
Speed (h)	14 ± 1	14 ± 1
XC skiing drills (h)	40 ± 6	34 ± 9
**Exercise modes**		
Running (h)	85 ± 3	81 ± 5^*^
Roller-ski skating (h)	11 ± 1	11 ± 2
Roller-ski classic (h)	8 ± 1	8 ± 2
Ski skating (h)	111 ± 3	108 ± 5
Ski classic (h)	70 ± 1	66 ± 7^*^
**Endurance training time**		
LIT (h)	232 ± 6	223 ± 12^*^
MIT (h)	13 ± 1	12 ± 1^*^
HIT (h)	26 ± 1	24 ± 2^*^
LIT/MIT/HIT (%)	85/5/10	86/5/9
**Training load**		
Load (sRPE/wk)	3825 ± 1013	3228 ± 748^*^
Load/volume ratio (sRPE/h)	4.9 ± 0.6	4.2 ± 0.5^*^

*LIT, low-intensity training; MIT, moderate-intensity training; HIT, high-intensity training*.

* Significant difference between groups (*P < 0.05).

**Figure 3:**
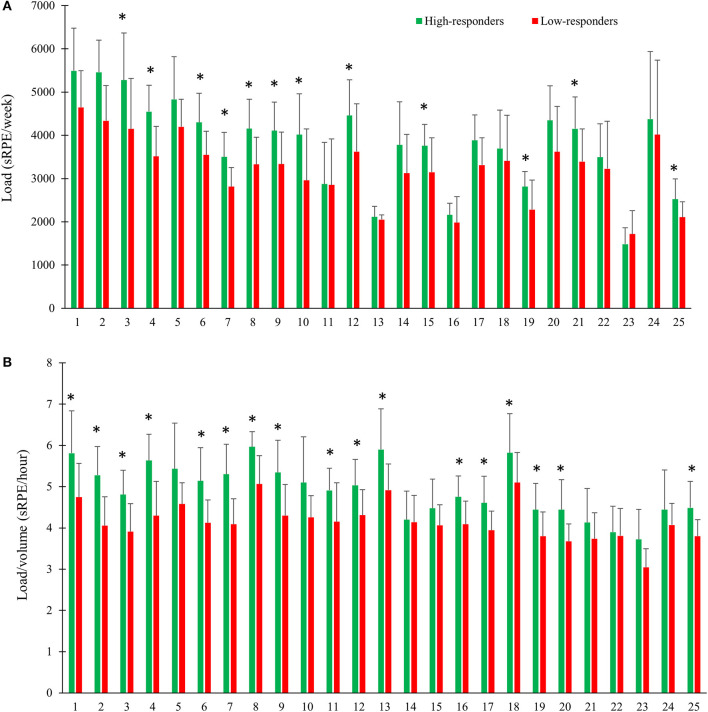
Quantification of **(A)** weekly training load and **(B)** weekly load/volume ratio in high- vs. low-responders during a 6-month XC ski-specific training period. *Significant difference between groups (*P* ≤ 0.05).

Weekly training load was significantly larger in high- compared to low-responders, and for most weeks the difference varied between ~8 and 25% (*P* ≤ 0.05; Figure 3A), with an average weekly training load for the 6-month period being 18% larger in high-responders (*P* ≤ 0.05; Table 2). When normalizing training load for training volume, we found ~8–20% higher load/volume ratio for most weeks in high-compared to low-responders (*P* ≤ 0.05; Figure 3B), with an average of 15% difference between groups (*P* ≤ 0.05; Table 2).

### Interviews

The main factors distinguish high from low-responders reported by the athlete's coaches are summarized in Tables 3, 4, including direct verbatim quotes. Based on these qualitative assessments, all coaches stated that high motivation to train and recover, and maintenance of motivation throughout the entire training period, were the most important factors distinguishing high- from low-responders. In addition, most coaches revealed higher passion for developing as a skier and the associated training process together with more enjoyment both during and between training sessions as the important characteristics distinguishing high- from low-responders (see Table 4). Lastly, most coaches stated that a stronger coach-athlete relationship characterized high-responders compared to low-responders, who also demonstrated more home sickness than high-responders.

**Table 3:** Multidisciplinary overview of physiological, technical and training related factors differentiating high- from low-responders following 6-months of XC ski-specific training in a group of endurance transfer athletes including direct verbatim quotes from the athlete's personal coaches.

**High-responders**	**Low-responders**	**Verbatim quotes from coaches**
**Physiological** • ↑*↑↑* VO_2max/peak_ • ↑*↑↑↑* Power output 4 mmol·L^−1^ roller-ski skating • ↓*↓↓↓* Submaximal heart rate roller ski-skating • ↑*↑↑↑* 1RM upper-body strength	**Physiological** • ↓ VO_2max/peak_ • ↑ Power output 4 mmol·L^−1^ roller-ski skating • ↓ Submaximal heart rate roller ski-skating • ↑↑ 1RM upper-body strength	“*The best responding athletes clearly developed their aerobic endurance capacity, whereas others showed no improvements and some even reduced their capacity, which is strange when following 6-months of dedicated endurance training.”* *“It seems like the best responding athletes were those with a sport background from running and I have a hypothesis that they developed their capacities more due to a better tolerance for high loads of endurance training within this exercise mode.”*
**Technical** • ↑*↑↑↑* Efficiency • ↑*↑↑↑* Cycle length • ↑*↑↑↑* On-snow technical skills^*^	**Technical** • ↑*↑↑* Efficiency • ↑*↑↑↑* Cycle length • ↑↑ On-snow technical skills^*^	“*The athletes who had high passion for skiing and enjoyed the learning process seemed to develop their technique the most.”* *“The ability to understand technical feedback and who worked dedicated with their given tasks over long time periods, even when not watched by their coach, was important for good technical development.”* *“The athletes who developed their endurance capacity the most, also demonstrated greatest technical development. This is because XC skiing is such a demanding endurance sport and you need to tolerate high training loads and perform a lot of repetitions, and at the same time maintain high training quality to develop your technical skills.”*
**Training** • ↑*↑↑↑* Total training volume & more sessions • ↑*↑↑* Volume of LIT & MIT & HIT • ↑*↑↑* Technique sessions (“XC skiing drills”) • ↑*↑↑↑* sRPE training load • ↑*↑↑↑*RPE during sessions	**Training** • ↓*↓↓* Total training volume & sessions • ↓*↓↓* LIT & MIT & HIT volume • ↓*↓↓* Technique sessions (“XC skiing drills”) • ↓*↓↓↓* sRPE training load • ↓*↓↓↓* RPE during sessions	“*In my opinion, it did not matter what changes in training load we did for some of the low-responding athletes, because they did not listen to and follow up according to our advices to similar extent as high-responders…”* *“The transfer to a new sport and exercise mode was more demanding than we thought, and we had to adjust the training load for athletes who were developing signs of overreaching. This applied for both groups, however, especially high-responders showed clear progress after reducing the load.”* *“Athletes with a high training response were motivated to ski and were really present during sessions and it felt like some of the less responding athletes sometimes were just not present or interested…”*

*VO_2max_, maximum oxygen uptake; VO_2peak_, peak oxygen uptake; sRPE, session rating of perceived exertion; RPE, rating of perceived exertion (1-10); 1RM, one repetition maximum*.

↑↓ *Effect size of pre- to post change within groups or difference between groups (↑/↓= trivial, ↑↑/↓↓= small, ↑↑↑/↓↓↓= moderate, ↑↑↑↑/↓↓↓↓= large)*.

* *Based on qualitative assessments of the athlete's personal coaches*.

**Table 4:** Multidisciplinary overview of health, psychological and sociological related factors differentiating high- from low-responders following 6-months of XC ski-specific training in a group of endurance transfer athletes including direct verbatim quotes from the athlete's personal coaches.

**High-responders**	**Low-responders**	**Verbatim quotes from coaches**
**Health** • ↓*↓↓↓* Incidents of injury and/or illness • Good health^*^	**Health** • ↑*↑↑↑* Incidents of injury and/or illness days • Athletes with signs of overtraining^*^	“*In total, there were few cases of injury or illness among high-responders, which might have been crucial for their better adaptations and development.”* *“High responders showed continuity in their work, maintained good health and found the optimal balance between load and recovery together with their personal coach.”*
Psychological^*^ • Highly motivated • Strong passion for skiing • Enjoyment during and between training sessions	Psychological^*^ • Less motivation • Less passion for skiing • Less enjoyment during and between training sessions	“*Motivation, enjoyment and passion, together with desire and curiosity to learn and improve… These are clear characteristics of the highest responding athletes… if you are not happy in life and with what you are doing, then it doesn't matter what you do and if you have the best coaches and the best training program… it doesn't matter…”* *“The best responding athletes gave things several tries and did not give up…they responded constructively to feedback, and showed an inner drive and interest to develop which can be hard to maintain in such a demanding project”*
Sociological^*^ • State of well-being individually and/or in the training group • Positive coach-athlete relationship	Sociological^*^ • Less well-being individually and/or in the training group • Less positive coach-athlete relationship • More homesickness	“*strong well-being in the process of transferring to a new sport and to a new country with different culture were important and homesickness were definitely more present among those athletes with a low training response”* *“high responding athletes communicated well with their coach and by that developed some level of independency/trust in their own work during the training period”* *“It seems like the boys liked better staying in Norway. They were tightly connected, had fun and spent awesome time together both during and between sessions”*

*↑↓ Effect size of difference between groups (↑/↓= trivial, ↑↑/↓↓= small, ↑↑↑/↓↓↓= moderate, ↑↑↑↑/↓↓↓↓= large)*.

* *Based on qualitative assessments of the athlete's personal coaches*.

## Discussion

The present study used a multidisciplinary approach to compare high- and low-responders following a 6-month sport-specific training period in endurance athletes transferring to XC skiing. High-responders improved VO_2max_ during treadmill running and VO_2peak_ when treadmill roller-ski skating from pre- to post (6 and 7%) more than low-responders who showed a corresponding small reduction (−1% and −3%). VO_2peak_ in double-poling ergometry did not change in any of the groups. Changes in skiing efficiency and cycle length did not differ between groups, although significant within-group improvements were found in both groups. The greater performance and endurance adaptations in high-responders were associated with higher training volumes (363 vs. 344 h), training loads (3825 vs. 3228), load/volume ratios (5 vs. 4) and lower incidents of injury and/or illness (5 vs. 10 days) in comparison to low-responders. In addition, qualitative interviews with their coaches highlighted that greater motivation together with the ability to build a strong coach-athlete relationship separated high- from low-responders.

### Development of Physiological and Technical Capacities

High-responders improved their running-VO_2max_ and VO_2peak_ in roller-ski skating, whereas low-responders displayed small reductions of these capacities over the 6 months of XC ski-specific endurance training. These changes were accompanied by larger improvements in power output at 4 mmol·L^−1^ and had reduced physiological strain at a given speed when submaximal roller-ski skating among high-responders. In contrast, the development of skiing efficiency and cycle length did not differ between-groups and were largely improved in both high- and low-responders. This is most likely explained by the low initial levels of both skiing efficiency and cycle length among these highly unexperienced skiers. Hence, there was a large potential for improving efficiency and cycle length among all athletes, while only high-responders were able to concurrently improve their energy delivery capacity (i.e., VO_2max_ and VO_2peak_). Overall, these findings demonstrate that high-responding transfer athletes can simultaneously develop their maximal/peak physiological capacities together with skiing efficiency and cycle length. This ability is highly important as concurrent development in both efficiency and VO_2max/peak_ is required for these athletes to reach the highest level in XC skiing (Sandbakk and Holmberg, 2017). In comparison, low-responders were able to develop their skiing efficiency and cycle length but did not improve their maximal/peak aerobic capacity from pre- to post.

The largest difference in performance and physiological development between groups where found for roller-ski skating, indicating that differences in technical development could have influenced the greater performance development in high-responders. Although the development of cycle length during submaximal roller-ski skating did not differ between groups, the coaches perceived that high-responders clearly developed their on-snow technical skills to a larger extent than low-responders. Thus, more sophisticated measures than those included in our approach could have detected these differences. In addition, moderate effect sizes indicated larger improvements in 1RM upper-body strength in seated pull-down and triceps-press among high-responders and could additionally have contributed to the larger sport-specific performance-development in high-responders by allowing them to produce more upper-body propulsion and thereby higher maximal speeds/power outputs during roller-ski skating and double-poling ergometry.

The greater improvements in peak physiological capacities when running and roller-ski skating among high-responders could be due to differences in sport background and/or gender, as we had relatively more runners and men in the group of high-responders. However, differences in sport background and gender were relatively equally distributed among low-responders (5/11 runners and 6/11 men) and based on the existing data, we can only speculate on this influence. Thus, future studies should aim to better understand the role of sport background when transferring athletes between sports and examine potential gender differences on endurance and performance adaptations following periods of standardized endurance training.

### Training and Training Load Characteristics

Even though standardized XC ski-specific training methods were utilized during the 6-month period, each athlete had a personal coach who helped them to daily adjust their training and ensure optimized training load and adaptations for each individual. Here, the outcome of this approach showed a larger training volume in high-responders, which is mostly explained by their higher compliance to training. This is likely associated with lower incidents of injury and illness as well as higher tolerance to training and thereby greater ability to maintain a high training load among high-responders.

The difference in total training time between groups during the 6-month period was only 6%, but the largest relative difference was found in the amounts of MIT and HIT performed (~8% difference). Since this training is suggested to provide effective cardiovascular stimulus it could have contributed to the larger developments of VO_2max_ and VO_2peak_ in high-responders (Buchheit and Laursen, 2013a,b). In addition, the greater perceived effort during sessions found in high-responders could imply a higher motivation to maintain training effort during the sessions and/or better ability to push themselves. This higher training tolerance might have elicited a greater overload stimulus and subsequently triggered better adaptations through a larger external load (e.g., average speed or distance) during training in this group of athletes.

The lack of adaptations in low-responders are most likely caused by this lower training tolerance, possibly leading to maladaptation and states of non-functional overreaching in some of the athletes with lowest responses, which are often associated with higher incidents of injury and illness (Budgett, 1998; Meeusen et al., 2013). This is further supported by feedback from the coaches, who reported that low-responders had less continuity in their training and more days of injury/illness. Furthermore, the coaches found it especially challenging to find the optimal training load among the low-responders who also struggled to perceive and communicate the load-recovery balance to their coaches (Foster et al., 2001). These findings highlight the importance of finding an optimal balance between load and recovery, while maintaining good health and avoiding injury and illness, to achieve good long-term development in endurance athletes. In addition, other factors than those measured in our design (e.g., genetic influence) could have contributed to explain parts of the observed variance in training response between the two groups (Mann et al., 2014).

### Psychological and Sociological Factors

The athlete's personal coaches reported motivation, passion, and a strong coach-athlete relationship to be the most important factors associated with a high-training response. While these two factors might have an independent influence on athlete development, their impact might also be interrelated. A strong coach-athlete relationship between high-responders and their respective coaches may have played an important role in optimizing the individual training loads and thereby positively influenced the adaptations of these athletes. However, a strong coach-athlete relationship might also have positively influenced the athletes' motivation and passion and vice-versa, and thereby reinforce the positive performance-development. For example, maintenance of motivation throughout the entire training period in high-responders might have contributed to their greater training effort and thus induced a larger overload stimulus and physiological development. Furthermore, higher passion for the training process and general well-being among high-responders might have enhanced the tolerance for and recovery from high training loads. In contrast, a less strong coach-athlete relationship combined with reduced performance-development in low-responders might have induced an imbalance between overall stress and recovery. Overall, these findings emphasize key psychological and sociological factors that coaches should consider when optimizing training adaptations and performance development in endurance athletes.

### Limitations of the Study

A potential limitation of our design was the lack of detailed background information of the athletes and their training characteristics during the period prior to the 6-months of XC ski-specific training. Another limitation was the relatively large variation in the duration of introduction period to roller-skiing before the pre-tests. However, we found no differences in the duration of the introduction period between high- and low-responders or significant correlations between the duration of the introduction period and pre- to post-test changes within groups or when all athletes were pooled. Lastly, classifying different responses to standardized periods of endurance training might be influenced by confounding factors such as technical measurement error and between-athlete variation (e.g., test-retest reliability) which should be considered in the interpretation of the results.

## Conclusion

This multidisciplinary comparison of high- and low-responders following a 6-month training period in endurance athletes transferring to XC skiing demonstrated greater improvements in running-VO_2max_ and roller ski-VO_2peak_ in high-responders compared to their lower responding counterparts. These superior physiological improvements were coincided by greater training compliance and less injury and illness during the training period. This allowed high-responders to achieve higher training loads and to tolerate greater perceived effort during sessions, which is the most likely reason for their greater improvements in maximal/peak aerobic capacity. Although skiing efficiency and cycle length were largely improved in both groups, there were no differences between high- and low-responders in this development. However, only the high-responding transfer athletes were able to concurrently develop their peak physiological capacities together with skiing efficiency and cycle length. Possibly, the higher motivation and stronger coach-athlete relationships in high-responders contributed to individually optimize their training and recovery routines, and thereby led to a more positive performance-development.

## Data Availability Statement

The raw data supporting the conclusions of this article will be made available by the authors, without undue reservation.

## Ethics Statement

The Regional Committee for Medical and Health Research Ethics waives the requirement for ethical approval for such studies. Therefore, the ethics of the study is done according to the institutional requirements and approval for data security and handling was obtained from the Norwegian Centre for Research Data.

## Author Contributions

RKT and ØS designed the study. RKT and XC performed data collection. RKT performed data and statistical analysis. RKT and ØS contributed to interpretation of the results. RKT and ØS wrote the draft manuscript. RKT, RvdT, XC, and ØS contributed to the final manuscript. All authors contributed to the article and approved the submitted version.

## Conflict of Interest

The authors declare that the research was conducted in the absence of any commercial or financial relationships that could be construed as a potential conflict of interest.
